# 

**Published:** 1855-02

**Authors:** 


### CONTENTS.

ORIGINAL COMMUNICATIONS :— PAGE

ART. I.—A List of the Principal Medicinal Plants enumerated by Botanists, and known to be growing in Northern Illinois and Wisconsin; with a slight notice of some of their medical properties. By Stephen Williams, M.D., . . . . . . . . . . 49

ART. II.—Case of Strangulated Hernia. Operation and Recovery. By Samuel Thompson, M.D., . . . . . . . . . 70

SELECTIONS :

Strychnine as a Poison—Trial of George W. Green for Murder, . . 75

BOOK NOTICES.

Transactions of the American Medical Association, . . . 87

EDITORIAL :

Small Pox, . . . . . . . . . . 95

Obituary Notice, . . . . . . . . . 96

